# Application and Optimization of Stiffness Abruption Structures for Pressure Sensors with High Sensitivity and Anti-Overload Ability

**DOI:** 10.3390/s17091965

**Published:** 2017-08-26

**Authors:** Tingzhong Xu, Dejiang Lu, Libo Zhao, Zhuangde Jiang, Hongyan Wang, Xin Guo, Zhikang Li, Xiangyang Zhou, Yulong Zhao

**Affiliations:** 1 State Key Laboratory for Manufacturing Systems Engineering, International Joint Laboratory for Micro/Nano Manufacturing and Measurement Technologies, Collaborative Innovation Center of SuzhouNano Science and Technology, School of Mechanical Engineering, Xi’an Jiaotong University, Xi’an 710049, China; tingzhongxu@163.com (T.X.); djlu@mail.xjtu.edu.cn (D.L.); zdjiang@mail.xjtu.edu.cn (Z.J.); aguoxinz@126.com (X.G.); zhikangli@xjtu.edu.cn (Z.L.); zhaoyulong@mail.xjtu.edu.cn (Y.Z.); 2 School of Mechanical and Manufacturing Engineering, University of New South Wales, Sydney, NSW 2052, Australia; 3 Shaanxi Institute of Metrology Science, Xi’an 710065, China; 4 School of Instrumentation Science & Optoelectronics Engineering, Beihang University, Beijing 100191, China; xyzhou@buaa.edu.cn

**Keywords:** bending stiffness distribution, peninsula-island structured diaphragm, stress concentration region, high sensitivity, high anti-overload ability

## Abstract

The influence of diaphragm bending stiffness distribution on the stress concentration characteristics of a pressure sensing chip had been analyzed and discussed systematically. According to the analysis, a novel peninsula-island-based diaphragm structure was presented and applied to two differenet diaphragm shapes as sensing chips for pressure sensors. By well-designed bending stiffness distribution of the diaphragm, the elastic potential energy induced by diaphragm deformation was concentrated above the gap position, which remarkably increased the sensitivity of the sensing chip. An optimization method and the distribution pattern of the peninsula-island based diaphragm structure were also discussed. Two kinds of sensing chips combined with the peninsula-island structures distributing along the side edge and diagonal directions of rectangular diaphragm were fabricated and analyzed. By bonding the sensing chips with anti-overload glass bases, these two sensing chips were demonstrated by testing to achieve not only high sensitivity, but also good anti-overload ability. The experimental results showed that the proposed structures had the potential to measure ultra-low absolute pressures with high sensitivity and good anti-overload ability in an atmospheric environment.

## 1. Introduction

Attributed to their low-cost and simple fabrication process, micro electromechanical systems (MEMS) piezoresistive pressure sensors had been widely applied in industry for several decades. Sensors with high sensitivity are often needed in a wide variety of fields. In addition to automotive applications such as tire pressure monitoring, hydraulic system fluid pressure sensing and engine manifold monitoring [1,2,3,4,5], pressure is also one of the most important physical parameters for various biomedical applications, including measuring intrauterine pressure during birth, monitoring the inlet and outlet pressures of blood in kidney dialysis and the cardiovascular system, measuring and controlling the vacuum level used to remove fluid from the eye during eye surgery [6,7,8,9], etc. One of the earliest research efforts in biomedical applications was a pressure sensor developed by Samaun et al. [10] for biomedical instrumentation applications.

Based on a pressure detection mechanism classification, the previous studies can be divided into three kinds of detection methods: resonant detection, deformation detection and stress detection methods. The resonant detection method is based on the principle that the pressure load influences the resonant frequency of a diaphragm structure. For example, Cheng et al. [11] presented a micro-pressure sensor based on center frequency detection of a double-ended quartz tuning fork attached on a diaphragm. Although the sensor had a sensitivity of 299 kHz·kPa^−1^ and a nonlinearity of 0.0278%FS, the quartz tuning fork fabrication process was not compatible with the MEMS technology. Li et al. [12] presented a pressure detection model based on an electrostatically actuated resonant microplate, which had a working range within 100 Pa in theory. However, its parasitic capacitance and the low quality factor causd by the array design lower its real performance.

The deformation detection method aims to detect the structure deflection induced by the pressure load, which is usually realized by a capacitive or optical sensing mechanism. For example, Chattopadhyay [13] presented a pressure sensor based on a capacitive sensing mechanism to detect the diaphragm deformation of the diaphragm, in which the sensor had a large non-linearity. Tang [14] presented an optical fiber grating-based pressure sensor with a sensitivity of −240 pm/MPa, however it had installation restrictions.

In addition, strain gauge and piezoelectric sensor can be based on the stress detection method. Piezoresistive sensors have higher piezoresistance coefficients and good accuracy with simple signal transduction characteristics [15,16,17,18,19,20,21,22,23,24,25,26,27,28]. Besides, their fabrication process as compatible with the current MEMS technology so they can be mass produced. Thanks to the advantages presented above, MEMS piezoresistive pressure sensors are the most widely applied nowadays.

As high sensitivity combined with low non-linearity are often very attractive performance features for a sensing chip, flat silicon diaphragms were generally modified with additional lump or boss structures to stiffen the diaphragm. These features improved the non-linearity by limiting the stretch deformation of the diaphragm, which is a significant cause of non-linearity [29]. However, the stiffened diaphragm also makes the diaphragm more constrained, lowering the measuring sensitivity of the sensors, so there is a contradiction between non-linearity and measuring sensitivity that cannot be solved easily. Previous studies usually focused on modifying the design of the lump or boss structures to resolve the contradiction [15,16,17,18,19,20,21,22,23,24,25,26,27,28]. For example, Hein et al. [26] used a structured diaphragm with four flexible beams and a rigid diaphragm centre to reduce nonlinearity effects. They presented piezoresistive micro sensors for the 300 Pa range with high sensitivity and excellent linearity. Bao et al. [18] presented a beam-diaphragm structure with a working range of 0–1 kPa. Although its non-linearity of 0.1%FS shown its good linearity performance, its sensitivity of 0.6901 μV/V/Pa was low. Yu et al. [23] introduced a high sensitive pressure sensor combined with a bossed diaphragm incorporated beam. It had a sensitivity of 11.098 μV/V/Pa, but its non-linearity of 3.046%FS was low. Huang et al. [22] introduced a peninsula structured diaphragm which remarkably lowered the non-linearity of a pressure sensor to 0.36%FS, but its sensitivity was only 3.68 μV/V/Pa. Besides the structures presented above for the measurement of low pressure, the low non-linearity and high measuring sensitivity of sensors was hardly improved at the same time in subsequent works [15,16,17,18,19,20,21,22,23,24,25,26,27,28].

In addition to the non-linearity and sensitivity performance, the anti-overload performance of a high sensitivity pressure sensor is also critical, especially in aerospace applications. Aircraft altitude can be determined by measuring pressure based on the relationship between absolute pressure and height. Because of the extremely low pressure at high altitude and a high overload caused by the atmosphere in the Earth environment, both high measuring sensitivity and good anti-overload ability are required. Hein [30] presented a capacitive pressure sensor for differential pressure with an overload capability without the need for an external overload protection. Johnson et al. [31] reported a ribbed and bossed structure. The incorporation of ribs into the diaphragm for stress concentration was proved to be effective in improving the sensitivity and non-linearity. However, the anti-overload ability was not good because of the thin bosses used. Yu et al. [25] proposed a beam-membrane-quad-island (BMQI) absolute pressure sensor with a sensitivity of 0.018 mV/V/Pa and non-linearity of 0.14%, but the quad-island structure necessary to withstand the overload severely impaired the 1st natural frequency of the sensing chip.

In this paper, a systematic design method for a beam-boss structured diaphragm based on the relationship between the diaphragm bending stiffness distribution and diaphragm stress concentration condition is presented for the first time. Secondly, giuided by the design method a novel peninsula-island-based diaphragm structure is presented and applied to different diaphragm shapes to test its performance. Thirdly, a three-step optimization method with good accuracy and generalization performance is presented. Finally, the sensing chips were bonded on an anti-overload glass base with a stepped structure, which guaranteed the proposed sensor had a good anti-overload ability.

## 2. Sensing Chip Design

A well designed diaphragm structure is critical to improve the performance of a sensing chip. The design of a diaphragm structure is closely related to its bending stiffness distribution on the diaphragm structure. In this part, a systematic analysis of the influence of diaphragm bending stiffness distribution on the stress concentration characteristics of a pressure sensing chip was conducted. Then, different diaphragms with peninsula-island structures were presented for further study.

### 2.1. Stiffness Distribution Characteristics of a Diaphragm Structure

The measuring sensitivity of a silicon-based piezoresistive pressure sensor is mainly determined by the stress concentration conditions in the stress concentration region (SCR) of the sensing chip. The more elastic potential energy converted to an electrical output through piezoresistors, the higher the sensitivity of the sensing chip will be.

High stress concentrations usually appear near a crack-like structure where the radius of curvature is the lowest. In an elliptical crack-like structure with length of 2*a*_0_ and width of 2*b*_0_, under an applied external stress *σ*, the stress at the ends of major axes can be given by Inglis’ equation [32]:

(1) $$\sigma_{\max}=\sigma \left(1+2\frac{a_{0}}{b_{0}}\right)=\sigma \left(1+2\sqrt{\frac{a_{0}}{\rho_{0}}}\right)$$

where *ρ*_0_ is the curvature radius of the crack tip. As the radius of curvature approaches zero, the maximum stress approaches infinity. However, this could not be utilized in the design of a sensing chip because the sensing chip structure can fail via a propagating crack, when a concentrated stress exceeds the material's theoretical cohesive strength. Also the irregular shape of SCR makes it impossible to fabricate piezoresistors on it. In order to form a SCR with high stress concentration and good stress value uniformity to improve the non-linearity of sensing chips, researchers usually design structures such as bossed diaphragms and diaphragms with bulky islands to redistribute the stiffness in different parts of the diaphragm. According to the previous diaphragm structures, the key points of diaphragm stiffness design can be concluded as follows:

- The stiffened diaphragm had the ability to concentrate elastic potential energy in SCR.
- The stiffened area should not constrain the diaphragm deformation too much which would impair the stress concentration.

However, the stiffened structure will definitely constrain the diaphragm deformation which may impair the stress concentration at the SCR, so a balance between these two key points must be found and make a full use of the first key point to enhance the stress concentration.

**Figure 1:**
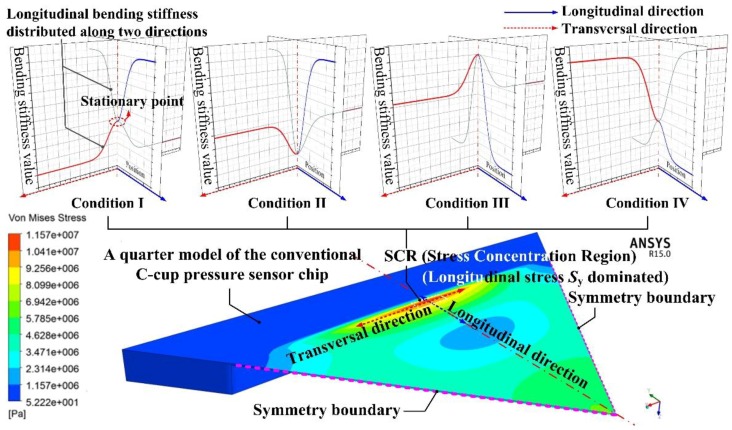
Stiffness distributions along two main directions around SCR of a pressure sensing chip.

Here, the emphasis was put on the stiffened structure design around the SCR. Considering the maximum stress usually appears at a stationary stiffness point on the diaphragm surface and the structural symmetric characteristic of the diaphragm, the stiffened structure around the SCR should be symmetric and the SCR center should be an intersection point of the stiffness stationary point along two main directions. With this in mind four different stiffness distribution conditions around the SCR can be conceived, as shown in Figure 1.

In condition I, the SCR had the lowest bending stiffness along the longitudinal direction and the highest bending stiffness along the transversal direction. Then, the SCR was the most deformable region along the longitudinal direction and the main role to resist the diaphragm deformation along the transversal direction.

In condition II, the SCR had the lowest bending stiffness along both the longitudinal and transversal directions, therefore, the SCR was the most deformable region along the two directions.

In condition III, the SCR had the highest bending stiffness along both the longitudinal and transversal directions. The SCR was a rigid body compared with the surrounding diaphragm structure.

In condition IV, the SCR had the highest bending stiffness along the longitudinal direction and lowest bending stiffness along the transversal direction. Then, the position on both sides along the transversal direction of the SCR played the main role to resist the diaphragm deformation.

#### 2.1.1. Condition I

**Figure 2:**
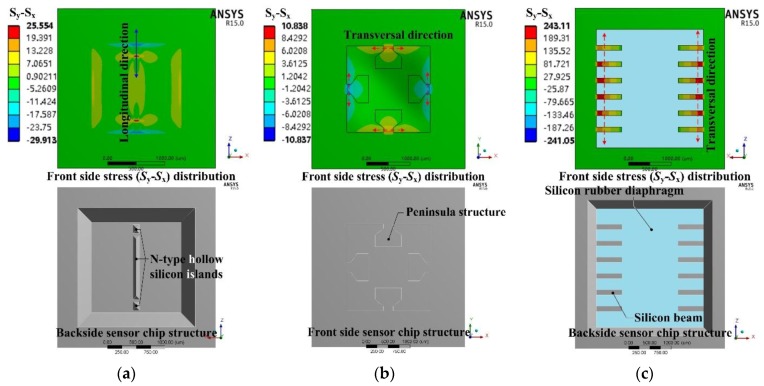
Diaphragm structure had either maximum bending stiffness along the transversal direction or minmum bending stiffness along the longitudinal direction: (**a**) Kinnell’s structure [21]; (**b**) Huang’s structure [22]; (**c**) Seo’s structure [33].

**Figure 3:**
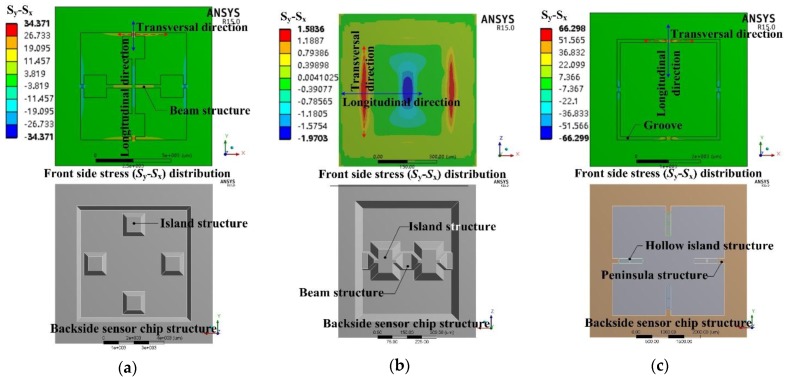
Diaphragm structures had both maximum bending stiffness along the transversal direction and minmum bending stiffness along the longitudinal direction: (**a**) Yu’s structure [25]; (**b**) Yang’s structure [34]; (**c**) Xu’s structure [35].

Most previously researched structures can be considered as belonging to condition I. However, these structures usually strictly satisfy the bending stiffness distribution only in one direction, which only has a maximum bending stiffness along the transversal direction, while the bending stiffness almost maintains a constant value along the other direction, and vice versa, like the structures shown in Figure 2. P.K. Kinnell [21] created a bending stiffness valley along the longitudinal direction by using n-type hollow silicon islands to stiffen the diaphragm. Huang [22] created a bending stiffness peak along the transversal direction by using a bossed peninsula structured diaphragm. Seo [33] created a bending stiffness peak along the transversal direction by a combination of a silicon beam and a silicon rubber diaphragm based on the large Young modulus difference between these two materials. For these structures, the elastic potential energy dissipation along the other direction without the bending stiffness stationary point was large. The sensing chip structures presented in [25,34,35] had both bending stiffness valleys along the longitudinal direction and bending stiffness peaks along the transversal direction realized by bossed beams, islands and groove structures, as shown in Figure 3. Compared with the structures presented in [21,22,33], the structures presented in [25,34,35] showed less elastic potential energy dissipation around the SCR boundary, so the stress concentration distribution of condition I can confined in a region surrounded by the junctions of the low bending stiffness area and high bending stiffness area. Also, based on the principle of energy conservation, a higher stress concentration can be obtained by shrinking the area of the SCR, by which means, the sensitivity of the sensing chip can be improved.

#### 2.1.2. Condition II

**Figure 4:**
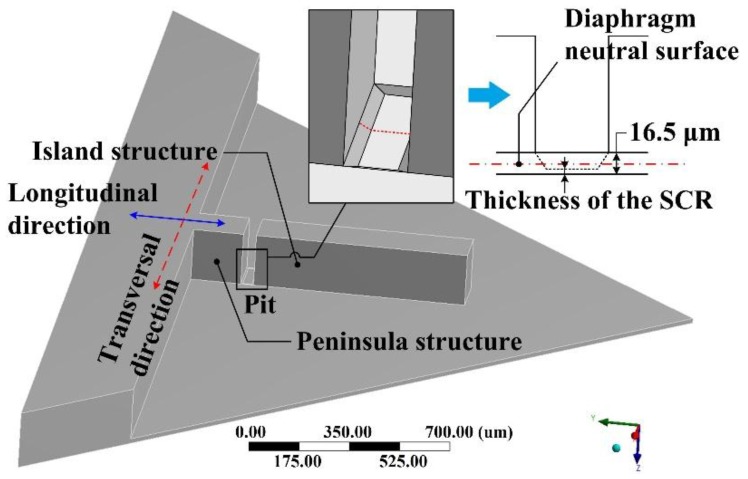
Diaphragm with peninsula-island structure and a pit.

Here a peninsula-island structured diaphragm with a pit at the SCR, as shown in Figure 4, was used to discuss the stress concentration characteristics of the condition II. The pit plays the role of the SCR. Also, the influence from the SCR thickness on the stress concentration in both the stress dissipation area and SCR has been discussed.

**Figure 5:**
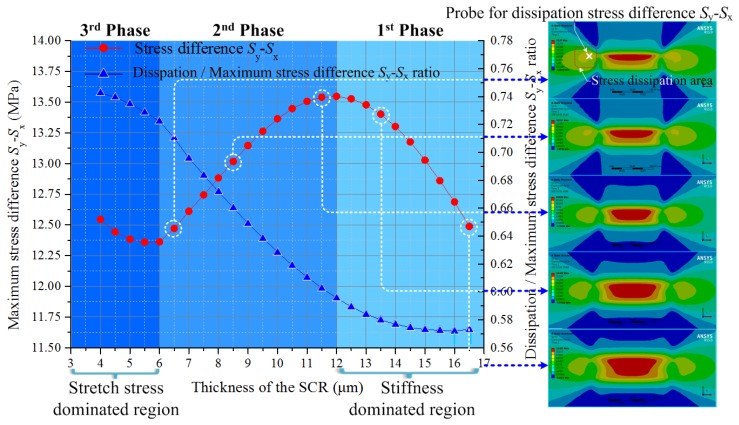
Stress concentration condition around the SCR influenced by the stiffness difference variation along the transversal direction.

As shown in Figure 5, by changing the SCR thickness, the stiffness difference between the SCR and the remaining region along the transversal direction can be divided into three phases.

In the first phase, the SCR thickness decrease lowered the bending stiffness of the diaphragm which increased the stress concentration value in both the stress dissipation area and SCR.

In the second phase, with the SCR thickness decreasing further, the bending stiffness difference between the SCR and the remaining part along the transversal direction became larger. The stress dissipation area with the higher bending stiffness acquired more elastic potential energy and this increased the stress concentration value [26]. Considering the principle of energy conservation, the stress concentration value at the SCR became lower.

In the third phase, with the SCR becoming even thinner, the stress concentration value at the SCR increased again. This is because the whole structure at the SCR was getting away from the neutral surface of the diaphragm, whereby the stress on the cross section of the SCR was dominated by stretching forces. The fast decrease in SCR cross section made the stress concentration at the SCR increase again.

However, according to the ratio between disspation stress and maximum stress difference shown in Figure 5, the dissipation stress increased during all three phases, which causes a lot of elastic energy dissipation. Also it is hard to realize and control the etching thickness for the necessary multilevel structure, so this kind of structure can hardly be used in pressure diaphragm structures.

#### 2.1.3. Condition III

In condition III, the SCR had the highest bending stiffness along both the longitudinal and transversal directions. The stress concentration value in this SCR can be derived by Euler-Bernoulli beam theory [36]. According to the Euler-Bernoulli beam theory, the maximum stress value was concentrated at the position around the SCR, instead of concentrating on the SCR with the highest stiffness, so condition III can’t be a stiffness distribution option for a piezoresistive pressure sensor.

#### 2.1.4. Condition IV

**Figure 6:**
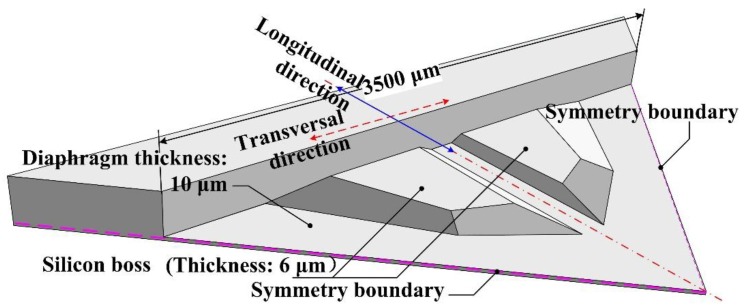
Stiffness distribution pattern for condition IV discussion.

**Figure 7:**
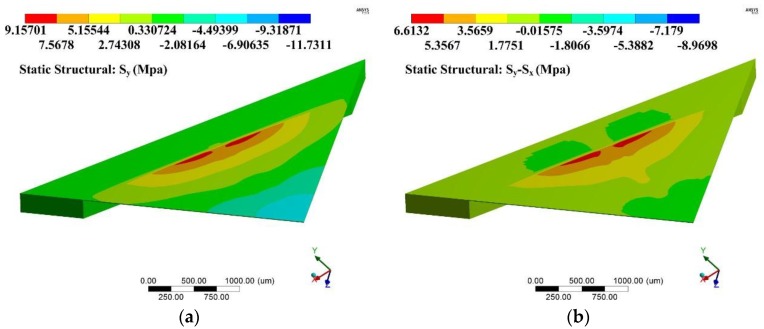
Stress distribution in condition IV: (**a**) longitudinal stress *S*_y_ distribution of the structure; (**b**) stress difference *S*_y_ − *S*_x_ distribution of the structure.

In condition IV, the SCR had highest bending stiffness along the longitudinal direction and lowest bending stiffness along the transversal direction. This kind of bending stiffness distribution can be realized by the structure shown in Figure 6. According to the stress distribution shown in Figure 7, the silicon boss region with higher bending stiffness plays the main role in resisting the deformation of the diaphragm, so the elastic potential energy is mainly concentrated at the region with higher bending stiffness along the transversal direction. Therefore, condition IV can’t be a stiffness distribution option for a piezoresistive pressure sensor.

In conclusion, conditions I and II can be potential options for bending stiffness distribution for a diaphragm structure. However, condition II always had a large elastic potential energy dissipation along the transversal direction which limits further improvement of the measuring sensitivity of the sensing chip, so condition I with the lowest bending stiffness along the longitudinal direction and the highest bending stiffness along the transversal direction was the best stiffness distribution for a piezoresistive pressure sensing chip. Based on previously researched peninsula-island structures [35,37,38], a peninsula-island structure with different distribution patterns and improved optimization method is presented as follows.

### 2.2. Basic Design of the Sensing Chip

**Figure 8:**
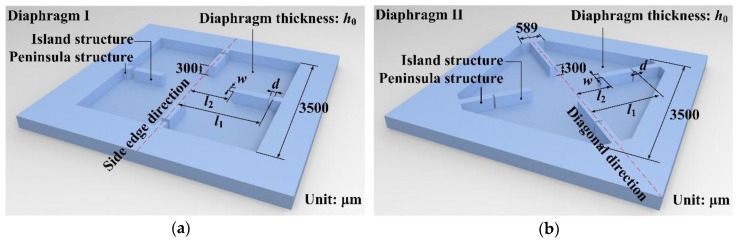
Schematics of the peninsula-island based diaphragms structures: (**a**) diaphragm I with peninsula-island structure positioning along the side edge direction of diaphragm; (**b**) diaphragm II with peninsula-island structure positioning along the diagonal direction of diaphragm.

As shown in Figure 8, two representative distribution patterns of a peninsula-island structure are presented, which are positioned along the side edge direction (Diaphragm I) and the diagonal direction of square diaphragm (Diaphragm II). Aiming for a working range of 500 Pa and considering the limitations of current fabrication technology, the thickness of the peninsula-island structure was around 300 ± 10 μm. The effective diaphragm dimension was set as 3500 × 3500 μm^2^.

## 3. Finite Element Method Analysis and Designing Method

### 3.1. The Effect of Various Geometrical Parameters on Stress and Frequency

**Figure 9:**
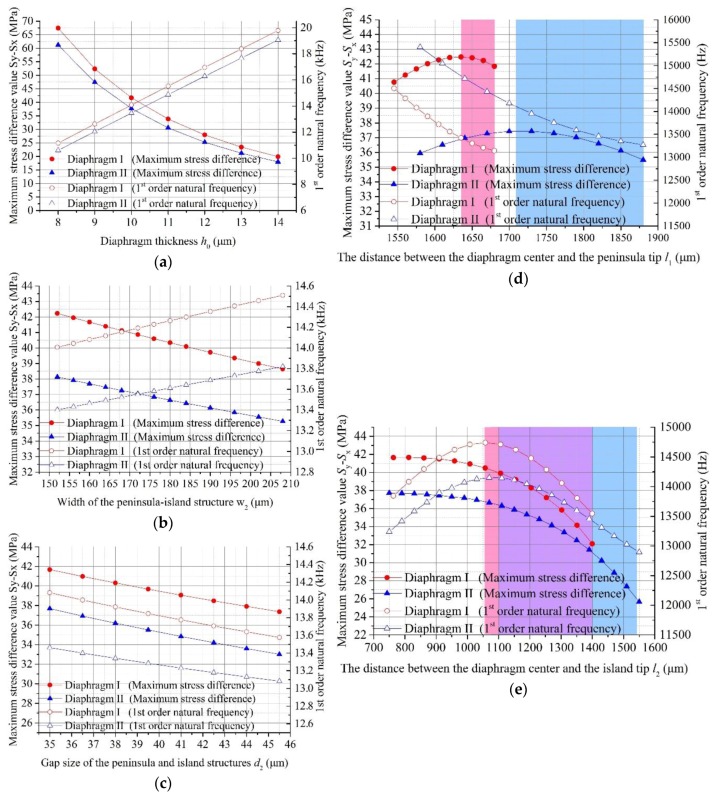
The effects of different geometrical parameters on *S*_y_ − *S*_x_ and *f*: (**a**) the effect of diaphragm thickness *h*_0_; (**b**) the effect of peninsula-island structure width *w*; (**c**) the effect of gap size *d*; (**d**) the effect of *l*_1_; (**e**) the effect of *l*_2_.

In this part, the influence of different geometrical parameters on the stress difference value (*σ* = *S*_y_ − *S*_x_) between the longitudinal and transversal stresses and 1st order natural frequency *f* are discussed, as shown in Figure 9.

The results indicate that the increasing stiffness of the whole diaphragm by thickening the diaphragm thickness *h*_0_ resulted in a decrease in *S*_y_ − *S*_x_ or an increase in *f*, as shown in Figure 9a. In Figure 9b, an increasing width *w* of the peninsula-island structure within a small optimization range can increase *f* by improving the partial stiffness of the diaphragm, while this can also increase the energy dissipation area to decrease the stress concentration at the SCR and *S*_y_ − *S*_x_. The results mentioned above shown that the tradeoff between *S*_y_ − *S*_x_ and *f* can’t be solved by optimizing the diaphragm thickness *h* and peninsula-island width *w*.

As shown in Figure 9c, as the dimension of the gap size *d* decreases the 1st order natural frequency and *S*_y_ − *S*_x_ increase at the same time. Also, as the coloured regions shown in Figure 9d,e show, the variations of the island and peninsula lengths can also made *S*_y_ − *S*_x_ and *f* increase at the same time within a certain optimization range. Compared with other structure sizes, the effects of *d*, *l*_1_ and *l*_2_ on the relationship between *S*_y_ − *S*_x_ and *f* are very different. Generally, the increasing stiffness leads to a decrease in sensitivity, while, *d* and a certain optimization range of *l*_1_, *l*_2_ made the *S*_y_ − *S*_x_ and *f* increase at the same time. Therefore, the tradeoff between the measuring sensitivity and dynamic performance of piezoresistive pressure sensing chip was remarkably relieved. As a result, these proposed diaphragm structures were able to remarkably improve the measuring sensitivity of a sensing chip without impairing its dynamic performance.

Diaphragm thickness is usually determined by the fabrication capacity and working range of a sensing chip; here the diaphragm thickness *h*_0_ was set as 10 μm. In order to improve the measuring sensitivity of the sensing chip, the SCR area should be small enough to diminish the elastic potential energy dissipation, so the SCR region was set as a rectangular region of 160 × 35 μm^2^ to spare enough space for arranging the piezoresistors. The *l*_1_ and *l*_2_ values are critical to optimize the sensitivity and dynamic performance of the sensing chip, as discussed in the following part.

### 3.2. Optimization Method for the Peninsula-Island Based Structure

**Figure 10:**
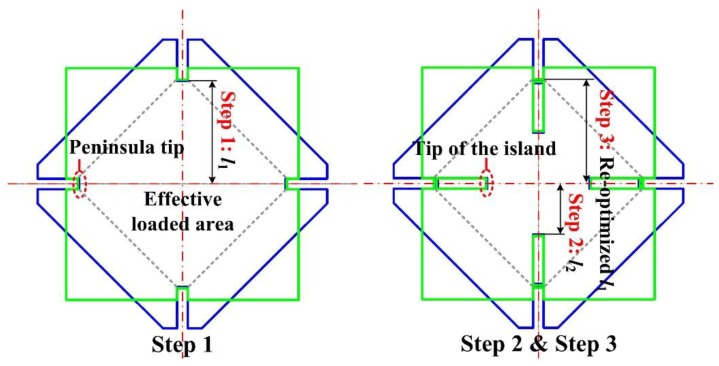
Optimization process of the peninsula-island structure for different distribution patterns.

For the proposed structures, the lengths of the peninsula and island structures are key parameters to optimize the measuring sensitivity. Based on the double-step optimization method presented in our previous research [38], a three-step method had been presented to improve the measuring sensitivity of sensing chips, as shown in Figure 10. The first step was to optimize the position of the peninsula tip without the island structure to relieve constraints arising from the silicon pedestal of the SCR. This was determined by the distance *l*_1_ between the peninsula tip and the diaphragm center. The second step was to optimize the distance *l*_2_ from the tip of island structure to the diaphragm center. This step maximizes the elastic potential energy transferred from the remainder of the diaphragm to the peninsula-island part of the diaphragm. The third step was to optimize the SCR position by redistributing the elastic potential energy transferred to the peninsula-island by the second step. In the third step, the lengths of the peninsula and island structures had been adjusted by *l*_1_ to guarantee the most elastic potential energy is concentrated in the SCR.

**Figure 11:**
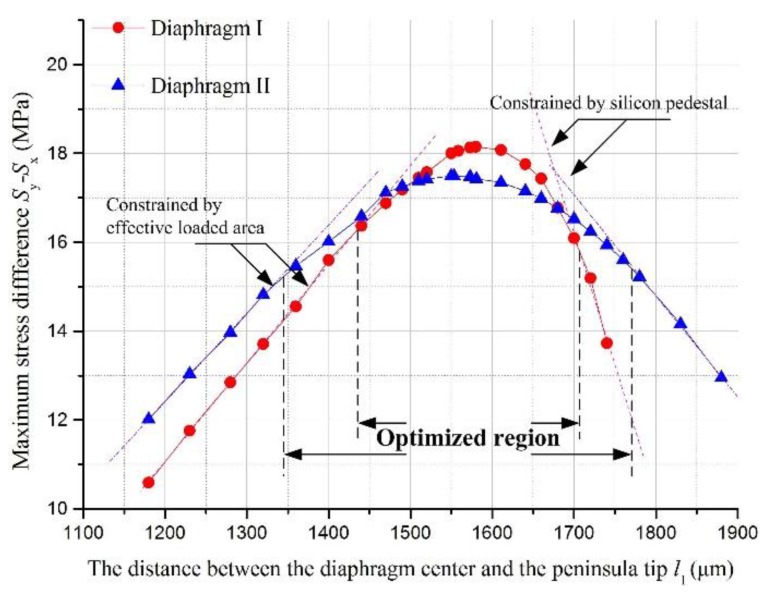
The relationship between maximum stress difference (*σ* = *S*_y_ − *S*_x_) and *l*_1_ for two diaphragm structures.

Figure 11 shown the results of the first optimization step. At two sides of the optimized range, the value of the stress difference at the SCR was constrained by the shrinkage of the effective loaded area and constraint force from the silicon pedestal, respectively. In these two areas, the stress difference decreased linearly with the change of *l*_1_ value. In this step, the SCR was set in a region with a lower constraint from the silicon pedestal. Also, an optimized range for the SCR position had been determined to provide a reference for *l*_1_ optimization in the third step.

**Figure 12:**
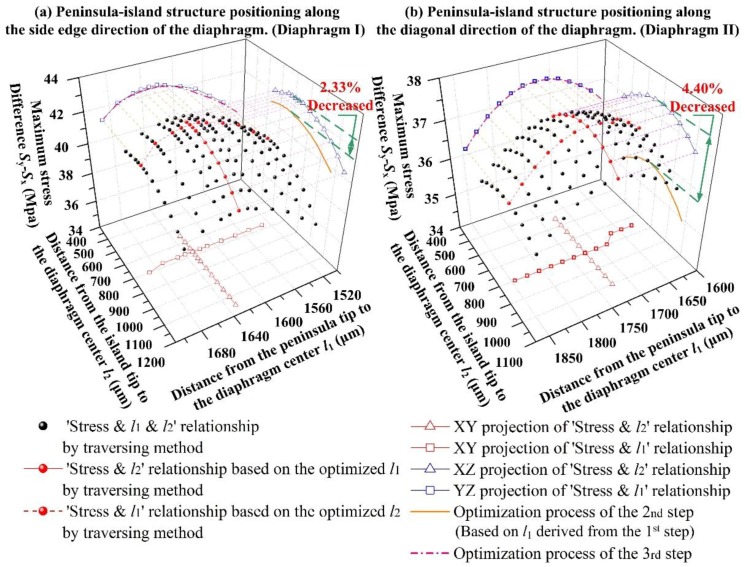
The relationship between stress difference (*σ* = *S*_y_ − *S*_x_) and *l*_1_ for two diaphragm structures.

In Figure 12, the traversing method was carried out to verify the results derived from the three-step method for both diaphragm structures. In the three-step method, the length *l*_2_ was determined by the second step and the length *l*_1_ was determind by the third step.

As shown in Figure 11 and Figure 12, the values of *l*_1_ for both structures deduced by the first step of three-step method were shorter than those deduced from the traversing method. The reason is that the peninsula length in the first step was optimized in a condition without an island structure. Then the stiffness of the structure without island structure was lower than that of structure with both peninsula and island structures. Compared with the diaphragm with higher stiffness, the stress concentration condition in the SCR was more likely to be influenced by the constraint from the silicon pedestal. In order to relieve the constraint from the silicon pedestal, the SCR needs to be set further away from the silicon pedestal. If the length *l*_1_ deduced from the first step were applied to the sensing chips, the stress difference at the SCR would be decreased by 2.33% for diaphragm I and 4.40% for diaphragm II, when compared to their optimized stress difference values with the length *l*_1_ deduced by the transversing method.

Based on the second step, by relocating the SCR position in the third step, the stress difference and optimized values of *l*_1_ and *l*_2_ agreed well with the results of the transversing method. The traversing method proved the feasibility of the three-step method which would greatly reduce the optimization workload.

Figure 12 also indicated that the optimized positions of the peninsula tip and island tip for the diaphragm I and diaphragm II were very close and the optimized stress difference values were not very sensitive to the size variation. Therefore, the optimized *l*_1_ and *l*_2_ for a diaphragm with certain dimensions could be determined, regardless of the distribution pattern of the peninsula-island structure.

**Figure 13:**
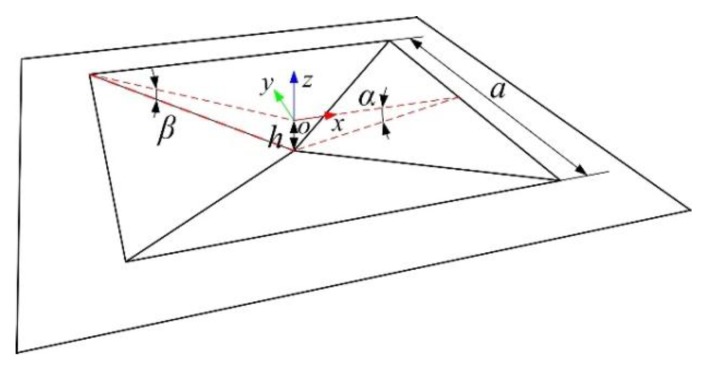
The relationship between stress difference (*σ* = *S*_y_ − *S*_x_) and *l*_1_ for two diaphragm structures.

However, the maximum value of stress difference was associated with the distribution patterns of the peninsula-island structure. The stress difference of diaphragm I was larger than that of diaphragm II. This can be explained by simplifing the deformation of a square diaphragm into the model shown in Figure 13 and given by Equations (2)–(4).

(2) $$\alpha =\arcsin\left(2h/\sqrt{4h^{2}+a^{2}}\right)$$

(3) $$\beta =\arcsin\left(\sqrt{2}h/\sqrt{2h^{2}+a^{2}}\right)$$

(4) $$\frac{a}{\beta }=\sqrt{\frac{a^{2}}{a^{2}+4h^{2}}+1}>1$$

where, *a* was the edge size of simplified model, *h* was the displacement at the center of simplfied model. Equations (2) and (3) shown the values of angles *α* and *β*. Equation (4) indicates that the value of *β* was definitly smaller than that of *α*. The stress value in the SCR was mainly caused by the bending moment which was directly propotional to the rotation angle of the diaphragm along the corresponding direction. This also indicated that the sensing chip with diaphragm I had a better performance to enhance the measuring sensitivity.

### 3.3. Stress Distribution Analysis of the Diaphragm

**Figure 14:**
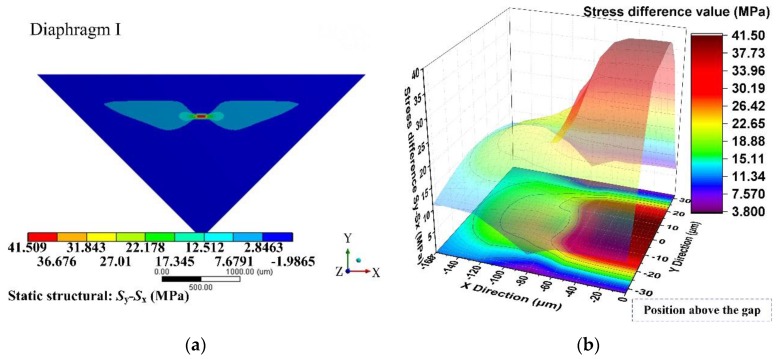
Stress difference (*σ* = *S*_y_ − *S*_x_) distribution of diaphragm I: (**a**) stress difference (*σ* = *S*_y_ − *S*_x_) distribution of a 1/4 model; (**b**) detailed stress difference (*σ* = *S*_y_ − *S*_x_) distribution of 1/2 SCR.

**Figure 15:**
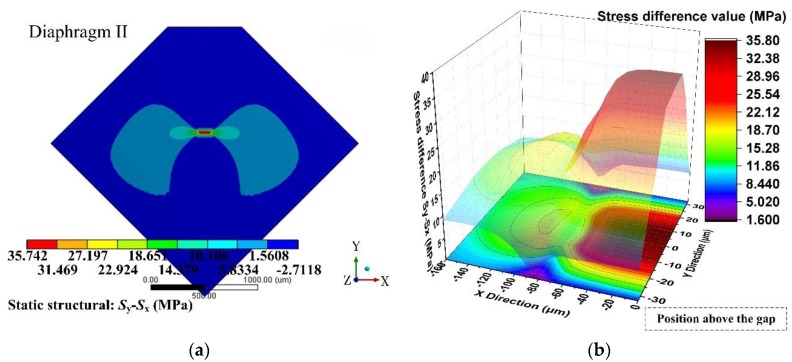
Stress difference (*σ* = *S*_y_ − *S*_x_) distribution of diaphragm II: (**a**) stress difference (*σ* = *S*_y_ − *S*_x_) distribution of a 1/4 model; (**b**) detailed stress difference (*σ* = *S*_y_ − *S*_x_) distribution of 1/2 SCR.

Based on the former optimization process, the performance of a sensing chip can be predicted using static analysis and modal analysis through the ANSYS software. Figure 14 and Figure 15 show the stress difference (*σ* = *S*_y_ − *S*_x_) distributions of the diaphragms under 500 Pa uniform loading. These figures indicated that the region above the gap had the largest stress difference value, which was a good verification for the stiffness distribution pattern presented in condition I. Apart from the region above the gap, there was no such stiffness abruption and no such elastic potential energy concentration characteristics.

## 4. Improved Diaphragm Structures

### 4.1. Structure Design

**Figure 16:**
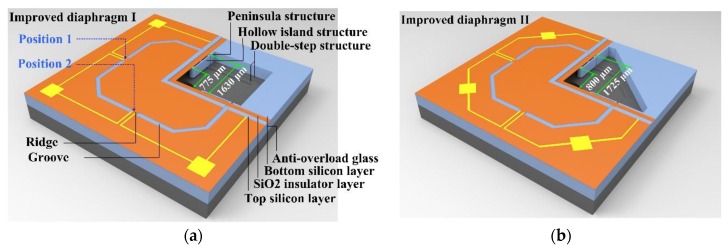
Schematics of the peninsula-island based bossed diaphragm structure: (**a**) improved diaphragm I with peninsula-island structure along the side edge direction; (**b**) improved diaphragm II with peninsula-island structure along the diagonal direction.

Based on the basic design, in order to create a stiffness peak along the transversal direction, the front side of diaphragm was designed as a bossed structure with four grooves. Here, considering a silicon on insulator (SOI) wafer (top silicon: 10 μm + buried SiO_2_: 1 μm) was used to realize the proposed structure, the grooves were set 5 μm deep and 109 μm wide. Four ridges were formed between each two grooves, as shown in Figure 16. The dimensions of the diaphragm and peninsula-island structure were both optimized according to Section 3.2.

### 4.2. Finite Element Analysis

**Figure 17:**
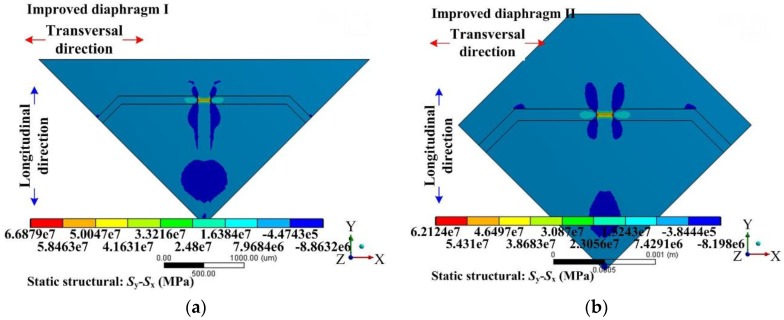
Stress difference (*σ* = *S*_y_ − *S*_x_) distributions of the improved diaphragms: (**a**) improved diaphragm I with peninsula-island structure along the side edge direction; (**b**) improved diaphragm II with peninsula-island structure along the diagonal direction.

Based on the longitudinal stiffness valley introduced by the peninsula-island structure discussed in Section 2.1.1. the evenly distributed grooves here not only eased the constraint from the silicon pedestal on the deflection of the diaphragm, but also created a stiffness peak along the transversal direction at the gap position shown in Figure 17. The ridge had the strongest stiffness compared with the groove region on both sides, and played a main role in resisting the diaphragm deformation. The longitudinal stress created by the diaphragm deflection would mainly concentrate at the SCR which was the top surface of the ridge. At the same time, the ridge formed a clear boundary between the SCR and other regions of the diaphragm along the transversal direction of the peninsula structure. The value of stress difference generated by the peninsula-island structure were further enhanced on the ridge and the elastic potential energy was strictly confined in the SCR, as shown in Figure 17.

**Figure 18:**
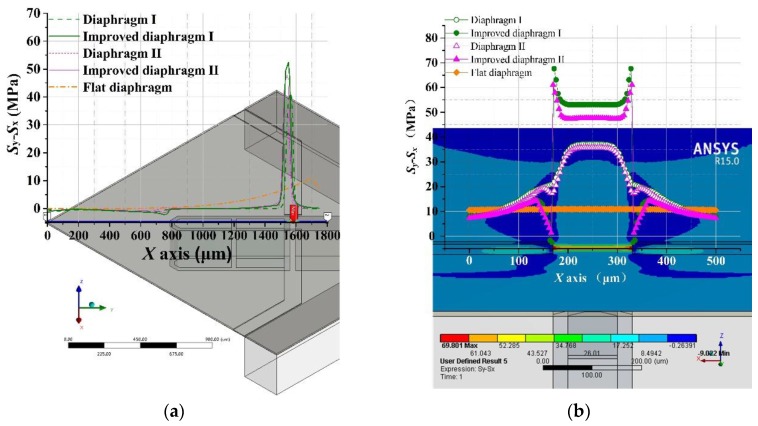

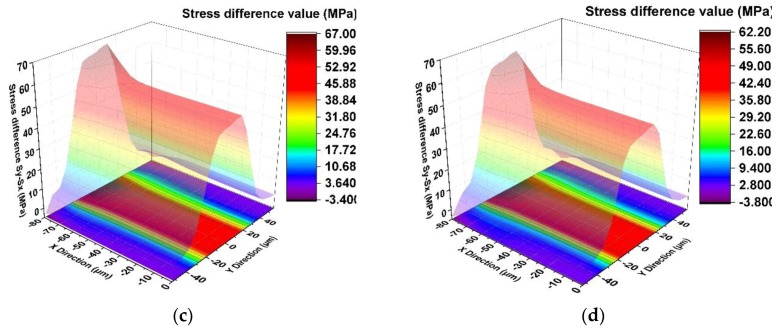
Stress distributions of different diaphragms: (**a**) stress differences (*σ* = *S*_y_ − *S*_x_) of different diaphragms from the central point to diaphragm edge; (**b**) stress differences (*σ* = *S*_y_ − *S*_x_) of different diaphragms in the SCR along the *X* direction; (**c**) stress difference (*σ* = *S*_y_ − *S*_x_) distribution of improved diaphragm I in the SCR; (**d**) stress difference (*σ* = *S*_y_ − *S*_x_) distribution of improved diaphragm II in the SCR.

The curves in Figure 18a represent and compare the stress difference distributions of different diaphragms from center to edge. The different diaphragms included the diaphragm I, diaphragm II, improved diaphragm I, improved diaphragm II and a flat diaphragm (C-cup diaphragm) with the same dimensions. It was evident that the stress differences of these proposed diaphragms reached their maximum values at the position above the gap. The improved diaphragm I had the maximum stress difference value, which was increased by 381% compared with the flat diaphragm without peninsula-island structure. As shown in Figure 18b, the stress difference (*σ* = *S*_y_ − *S*_x_) of flat diaphragm without peninsula-island structure changed slightly along the transversal direction. Remarkably, for the peninsula-island based bossed diaphragms structure, the stress differences (*σ* = *S*_y_ − *S*_x_) in their SCR were much larger than those of any other diaphragms.

For the proposed diaphragm structures, the stress distribution characteristics at the SCR were studied in detail. Considering the symmetry of structure, the stress distributions of only half SCR were shown in Figure 18c,d, which indicate that the center region of the ridge is the best region to arrange the piezoresistor, where the stress difference is not only very uniform but also large enough.

**Figure 19:**
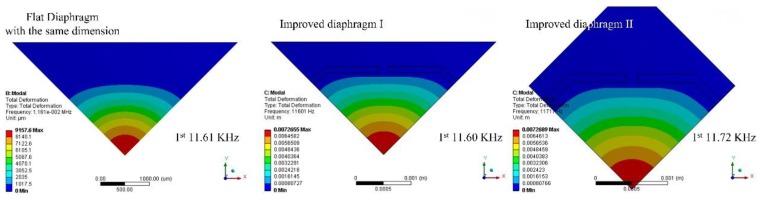
Dynamic behaviors of flat diaphragm, improved diaphragm I and improved diaphragm II.

Besides the sensitivity and linearity performance, the mechanical stability of a diaphragm is also important for high sensitivity pressure sensors used in high accuracy measurements. The mechanical stability of the diaphragm is closely related to its 1st order natural frequency. In order to improve robustness of the diaphragm, a higher 1st order natural frequency is preferred. Compared with the flat diaphragm, the 1st order natural frequencies of the proposed diaphragms almost remained the same, as shown in Figure 19, and much higher than those of beams-membrane-mono-island (BMMI) [23] and beam-membrane-quad-island (BMQI) [25] with the same working range of 500 Pa.

## 5. Anti-Overload Design

**Figure 20:**
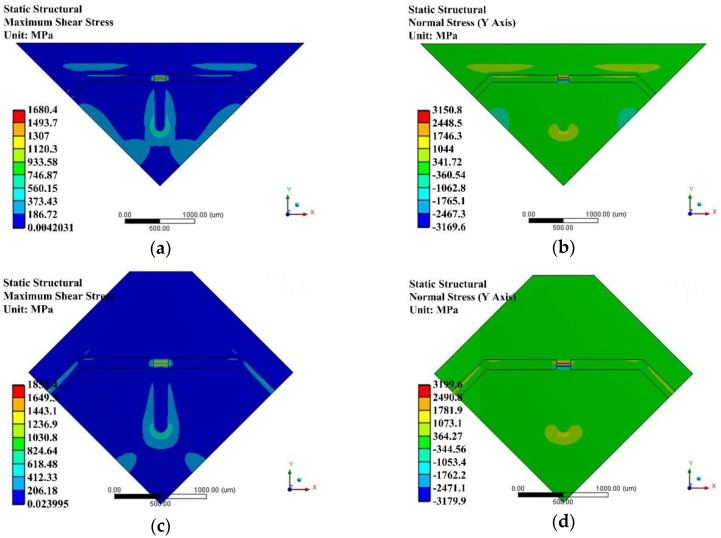
Stress distributions under overload condition based on conventionally designed anti-overload glass base under 1 atm: (**a**) shear stress *γ* of improved diaphragm I; (**b**) tension stress *S*_y_ of improved diaphragm I; (**c**) shear stress *γ* of improved diaphragm II; (**d**) tension stress *S*_y_ of improved diaphragm II.

Conventionally, anti-overload structures were usually based on the properly designed space between the glass base and the mass block attached to the back side of a diaphragm [23,25]. However, the mass block may stick to the glass base and be unable to return to its working status, because there exists intermolecular attraction and electrostatic attraction between the large area contact surfaces, and even if an anti-absorption electrode is fabricated on the contact surface of the glass base this can hardly prevent the mass block from adhereing to the glass base. Besides, the conventionally designed anti-overload structures may cause a huge shear stress in the SCR under 1 atm loading condition, which would destroy the structure, as shown in Figure 20. Based on the conventional anti-overload structure, the maximum tension stress and maximum shear stress would be concentrated in the SCR. Even worse, the values of the maximum shear stresses in the SCR both exceed the silicon shear strength (1.3 GPa) [39].

**Figure 21:**
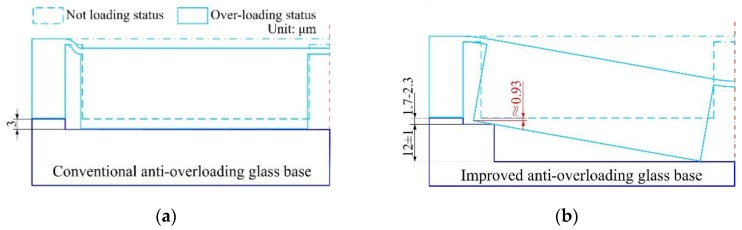
Working mechanism comparison between the conventional and stepped structures of anti-overload glass bases: (**a**) conventional anti-overloading design; (**b**) improved anti-overload design.

In order to avoid the over-critical shear stress and adhesion problem between the mass block and glass base, a stepped structure is proposed for the anti-overload glass base. The stepped structure enables the island structure to remain a sloping state when undergoing an overload condition, as shown in Figure 21. By this means, it converts a shear stress dominating condition to a tension stress dominating condition, which is mainly caused by the bending moment in the SCR. Also the stepped structure of the anti-overload glass converts a conventionally face to face contact form to the line to face contact form, which remarkably reduces the contact area and solves the absorption problem during overloading conditions.

**Figure 22:**
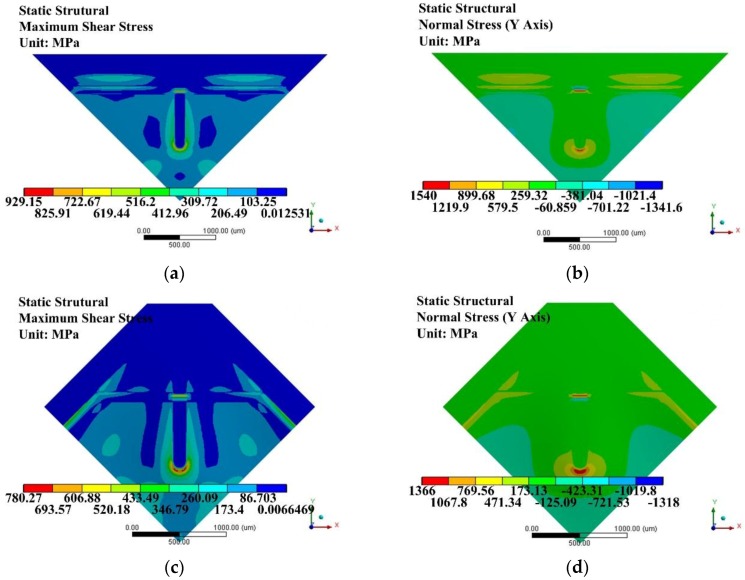
Stress distributions under overload condition based on anti-overload glass base with stepped structure under 1 atm: (**a**) shear stress *γ* distribution of improved diaphragm I; (**b**) tension stress *S*_y_ of improved diaphragm I; (**c**) shear stress *γ* of improved diaphragm II; (**d**) Tension stress *S*_y_ of improved diaphragm II.

Figure 22 indicates that the stepped structure of the anti-overload glass base was very effective in reducing the maximum stress in the SCR when exposed to overloading conditions. Compared with the conventional one, the maximum values of shear stress of both bossed diaphragms were reduced below 1 GPa. This reserves a sufficient safety factor for the proposed diaphragms.

## 6. Performance Experiments

**Figure 23:**
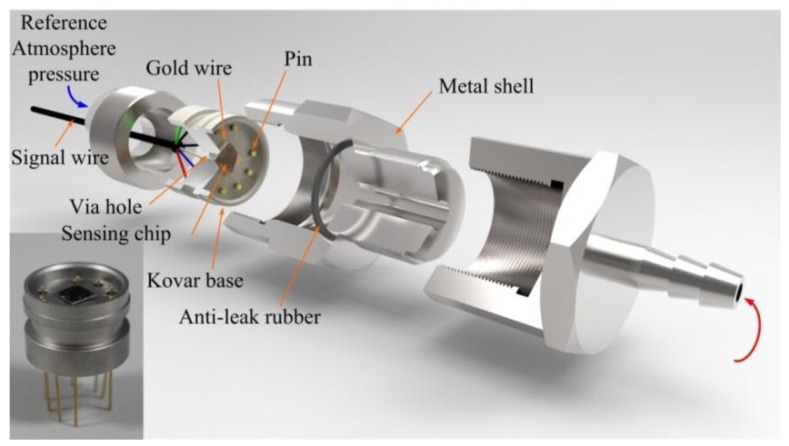
Schematic of the packaged pressure sensor.

Here the pressure sensors using different sensing chips with peninsula-island bossed diaphragms as shown in Figure 16 were fabricated to verify the design theory. The detailed fabrication process of the sensing chip was described in the Appendix [35]. The schematic packaging structure of the pressure sensor is shown in Figure 23. The sensing chip was attached to a Kovar base. The electrodes on the sensing chip were connected to the pins by gold wire bonding. The pins were connected to the signal wire from the backside of the Kovar base. Then, the Kovar base was assembled in a metal shell to test the sensor performance.

### 6.1. Anti-Overload Experiment

**Figure 24:**
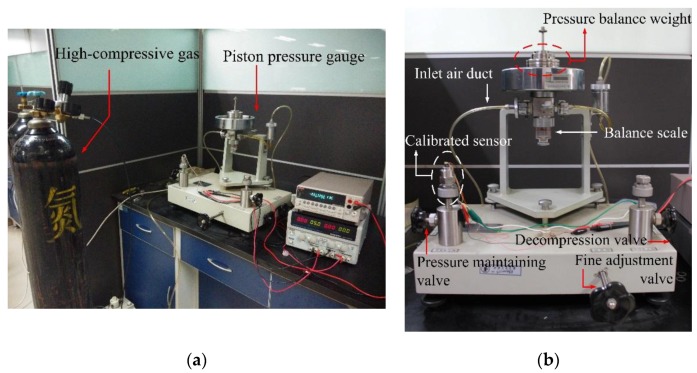
Experiment facilities for the anti-overload test: (**a**) the photo of whole set; (**b**) piston pressure gauge.

**Figure 25:**
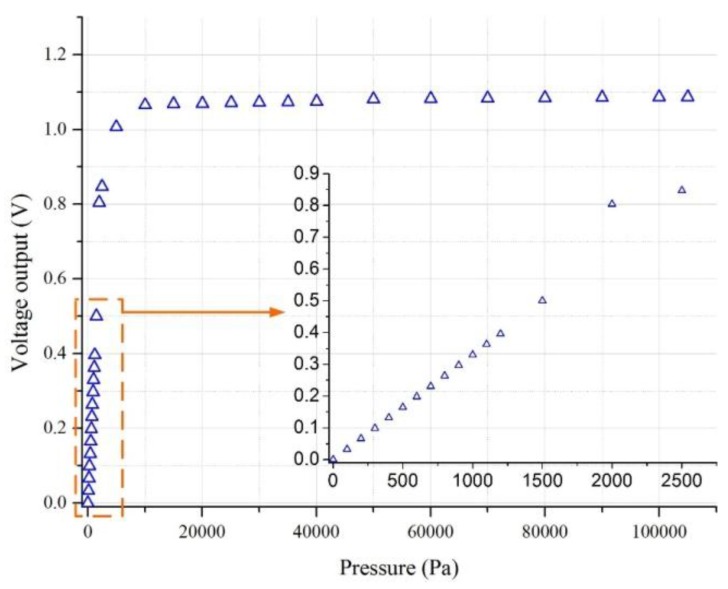
Output voltage versus overloading pressures up to 105 kPa for improved diaphragm I.

In this test, the anti-overload ability of the improved diaphragm I was tested using a piston pressure gauge (CWZ-4T) which can apply a maximum standard pressure with the value of 400 kPa, as shown in Figure 24. Firstly, a highly-compressive gas was used to apply an initial pressure of around 500 kPa to the piston pressure gauge. Secondly, pressure balance weights were used to set the test pressure. Thirdly, the decompression valve and fine adjustment valve were used to balance the applied pressure, and then applied the standard pressure to the calibrated sensor. In this process, the applied pressure was increased gradually at a pressure interval of 10 kPa until it increased to 105 kPa, which meant the sensor had an overload ability of 21,000%FS. The output voltage is shown in Figure 25 when the applied pressure was up to 105 kPa. Based on the output voltage, the island structure contacted the anti-overload glass base when the pressure reached to be around 2200 Pa. After several test rounds, the sensing chip was continued to be validated in the following sensitivity experiments, which demonstrated the sensing chip was safe and sound after overloading.

### 6.2. Sensitivity Experiment

**Figure 26:**
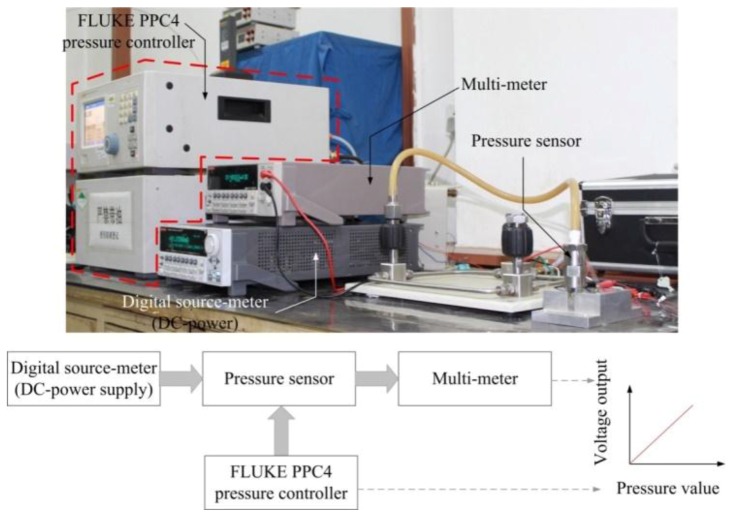
The pressure calibration facilities used in sensitivity experiment.

The sensitivities of the pressure sensors were tested by the experimental facilities shown in Figure 26. The pressure was loaded onto the sensing chip from the PVC hose using a pressure controller (FLUKE PPC4). The temperature performance of the pressure sensors was tested in a constant temperature oven (ESPEC PG-2J) from 20 °C to 60 °C.

**Figure 27:**
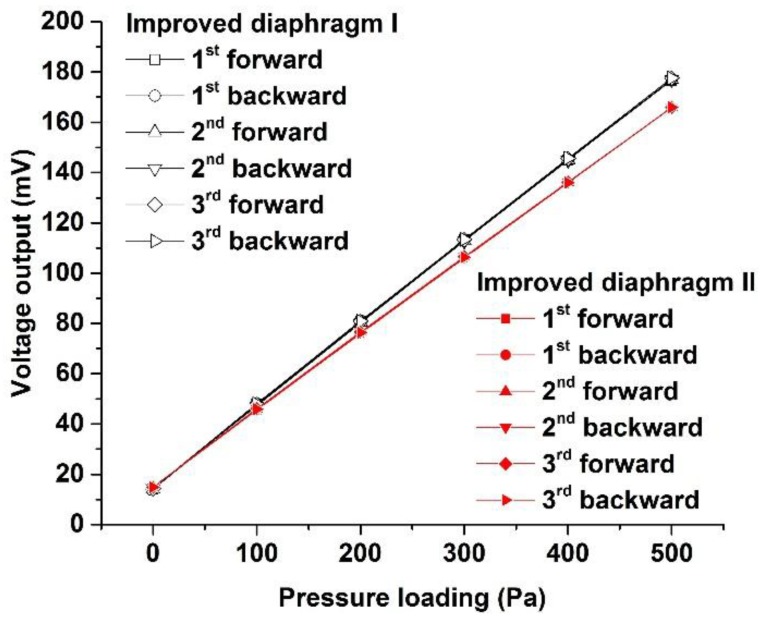
Output voltage versus applied pressure with a range from 0 to 500 Pa.

The calibrated data of three cycles of loading and unloading pressure for two developed sensors, as shown in Table 1 and Table 2, were plotted and fitted by the least squares method, as shown in Figure 27.

### 6.3. Zero Drift Experiment

**Figure 28:**
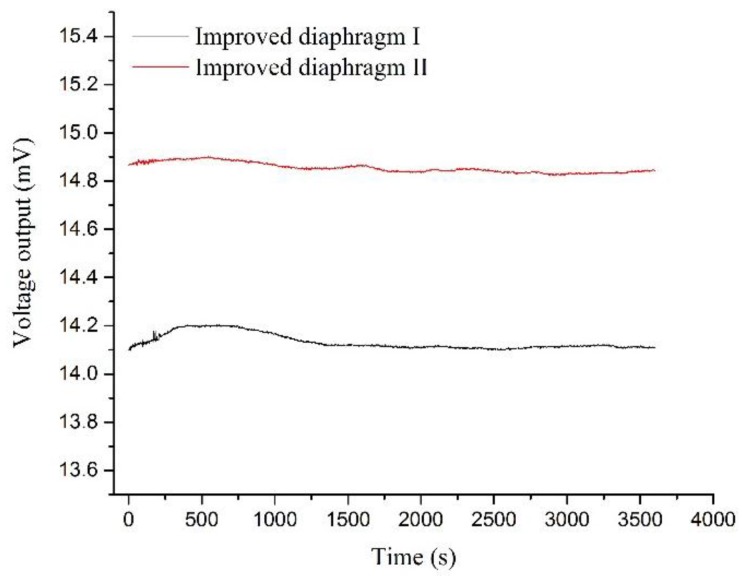
Zero drift data for two proposed sensing chips.

Zero output was mainly caused by the piezoresistor fabrication quality and residual stress caused by the sensing chip packaging process. The equation for zero drift can be presented as:

(5) $$Z=\frac{\Delta V_{0\max}}{V_{FS}}\times 100\%$$

where ∆*V*_0max_ is the difference between the initial voltage output and the maximum or minimum voltage out during the testing time; *V_FS_* was full scale output of the sensing chip. The sensing chip with two different diaphragm shapes were powered by 5 V DC voltage. The sensing chips were placed in 20 °C room temperature. The tests lasted for an hour and the data-collection interval was one second. The zero drift results for two different diaphragms are plotted in Figure 28. The zero drift values for the improved diaphragm I and II were 0.064%FS and 0.024%FS, respectively.

The performance of the proposed sensor chips is presented in Table 3. The performance comparision with some other typical low pressure sensing chips is presented in Table 4. According to the comparision data listed in Table 4, the proposed sensing chip had the best sensitivity and good nonlinearity performance. Also the 1st natural frequencies of the proposed sensing chips were the highest among the compared 500 Pa working range sensing chips.

## 7. Conclusions

This paper provided a systematic analysis of the influence of the diaphragm stiffness distribution on the stress concentration characteristics of a pressure sensing chip, which provides a guideline for diaphragm design for piezoresistive pressure sensing chips. Based on our systematic analysis, the optimization method and distribution patterns of peninsula-island structure were also discussed to improve the performance of sensing chips. An anti-overload glass base with stepped structure guaranteed a sensor with a high anti-overload ability of 21,000%FS. In accordance with the FEM results, the experimental results showed that the fabricated pressure sensors using bossed diaphragms combined with side edge and diagonal directional positioned peninsula-islands had sensitivities of 0.065 mV/V/Pa and 0.060 mV/V/Pa, respectively, and nonlinearity errors of 0.33%FS and 0.30%FS, respectively, within the pressure range of 0–500 Pa. It was concluded that pressure sensors with the proposed bossed diaphragms had excellent sensitivity, linearity and stability. The pressure sensors with the proposed diaphragm are potentially a better choice to measure ultra-low pressures in the fields of biomedical instruments, smart homes and aerodynamics

## Figures and Tables

**Table 1:** Calibration data for the improved diaphragm I.

Pressure (Pa)	Voltage Output (mV)							
	1st Round		2nd Round		3rd Round		Mean Value	
	Forward	Backward	Forward	Backward	Forward	Backward	Forward	Backward
0	14.10	14.40	14.10	14.50	14.10	14.40	14.10	14.43
100	47.87	48.13	47.48	47.22	47.75	47.93	47.70	47.76
200	80.91	81.12	80.5	80.21	80.75	80.95	80.72	80.76
300	113.22	113.32	112.9	112.74	113.33	113.46	113.15	113.17
400	145.03	145.27	145.1	144.9	145.52	145.74	145.21	145.30
500	176.73	176.73	177	177	177.57	177.57	177.10	177.10

**Table 2:** Calibration data for the improved diaphragm II.

Pressure (Pa)	Voltage Output (mV)							
	1st Round		2nd Round		3rd Round		Mean Value	
	Forward	Backward	Forward	Backward	Forward	Backward	Forward	Backward
0	14.80	14.90	14.85	14.97	14.8	14.84	14.82	14.90
100	45.53	45.56	45.98	46.10	46.05	45.87	45.85	45.84
200	75.94	76.03	76.54	76.61	76.59	76.32	76.36	76.32
300	106.02	106.12	106.55	106.56	106.5	106.33	106.36	106.34
400	135.91	136.00	136.22	136.36	136.24	135.97	136.12	136.11
500	165.90	165.85	165.91	165.90	166.00	166.00	165.94	165.92

**Table 3:** The sensor performance of proposed sensing chips.

Parameter	Improved Diaphragm I	Improved Diaphragm II
Reference temperature (°C)	20	20
Supply voltage (V)	5	5
Full scale output (mV)	165	151
Sensitivity (mV/V/Pa)	0.065	0.060
Nonlinearity (%FS)	0.33	0.30
Hysteresis (%FS)	0.36	0.16
Repeatability (%FS)	0.67	0.26
Accuracy (%FS)	0.94	0.55
Zero drift (%FS)	0.064	0.024

**Table 4:** The sensor performance comparisons among the proposed bossed diaphragms and previously researched sensing chips.

Diaphragm Structure	Sensitivity (mV/V/Pa)	Nonlinearity (%FS)	1st Natural Frequency (kHz)	Working Range (Pa)
Improved diaphragm I	0.065	0.33	11.6	500
Improved diaphragm II	0.060	0.3	11.7	500
BMMI [23]	0.011	3.05	7.0	500
BMQI [25]	0.018	0.124	10.2	500
C-cup with the same dimension	0.013	0.8	11.6	500
Beam-diaphragm structure [18]	0.00069	0.1	/	1000
Peninsula structured diaphragm [22]	0.00368	0.36	44.2	5000
CBM [24]	0.00154	0.19	44.5	5000
Hollow stiffening structure [21]	0.0116	0.4	/	3000

**Figure A1:**
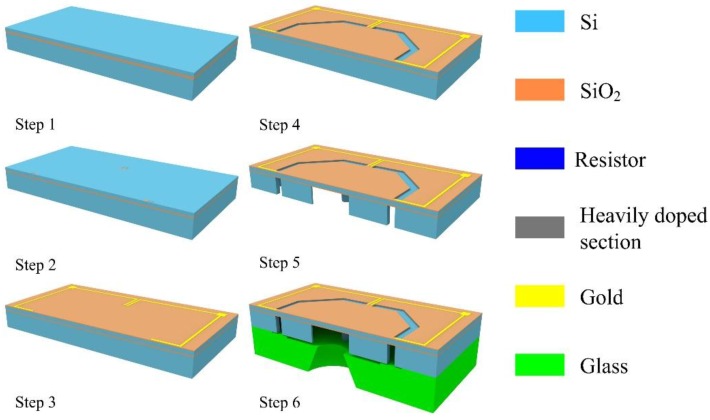
Main fabrication process of sensing chip.

**Figure A2:**
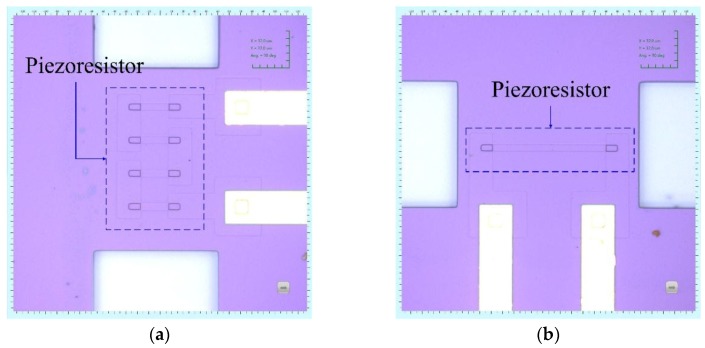
Microscopic photos of piezoresistors in two patterns: (**a**) piezoresistor in position 1 shown in Figure 16; (**b**) piezoresistor in position 2 shown in Figure 16.

**Figure A3:**
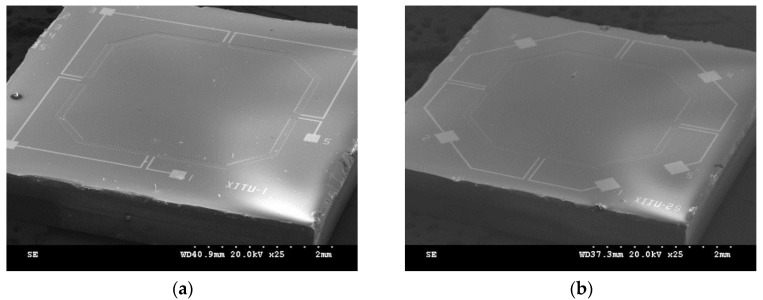
SEM pictures of fabricated sensing chips with different bossed diaphragms: (**a**) improved diaphragm I; (**b**) improved diaphragm II.

**Figure A4:**
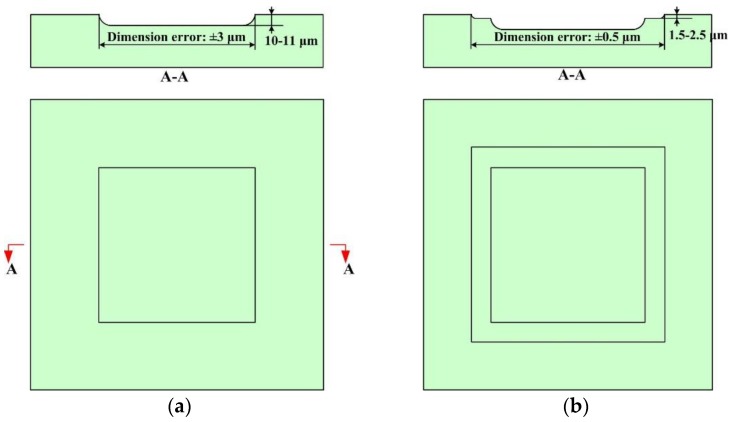
Fabrication process for stepped glass base: (**a**) 1st step for glass base etching; (**b**) 2nd step for glass etching.

## Abbreviations

The following abbreviations are used in this manuscript:

MEMS	Micro-electromechanical Systems
SCR	Stress Concentration Region

## Appendix A

MEMS technologies are employed to fabricate the sensing chip. The main processes were listed as follows, as shown in Figure A1.

Step 1. The starting material is a n-type (100) double-sided polished single-crystal silicon on insulator (SOI) material fabricated by separation by implantation of oxygen (SIMOX) technology. The thicknesses of top silicon layer, buried SiO_2_ layer and bottom silicon layer are 10 μm, 1 μm, 300 μm, respectively.

Step 2. Considering high piezoresistive coefficient and low temperature coefficient of p-type silicon, the implantation and diffusion of B (boron) is performed from a B_2_O_3_ constant source into the top Si layer followed by annealing process to eliminate some crystal lattice defects and improve the conducting power of top Si layer. After the thermal activation process, four piezoresistors are achieved with sheet resistance of 220 Ω/Υ, as shown in Figure A2. The initial resistance values of four piezoresistors are all around 3.8 kΩ.

Step 3. The SiO_2_ layers are formed onto top Si layer and bottom of substrate with oxidation process in O_2_. The resistor holes are etched by reactive ion etching (RIE). Gold is sputtered on the top of SiO_2_ layer to connect four piezoresistors into a Wheatstone bridge. Then, thermal treatment for ohmic connection and metallization is carried out in vacuum.

Step 4. Four grooves on top silicon structure are fabricated by RIE. Then, a ridge is formed between each two grooves.

Step 5. The back cavity of structure is etched by deep reactive ion etching (DRIE) to form the center effective membrane and the peninsula-island structure.

Step 6. Finally, the wafer is packaged on the steped glass with the thickness of 0.4 mm by anodic bonding technology in vacuum, and then diced into single sensing chips with bonded glass by scribing technology. Figure A3 shows the fabricated piezoresistive sensing chips.

And the stpped glass base was fabricated by isotropic etching by HF. First, use the HF to etch a cavity with 10–11 μm depth, and error for the pattern dimension was ±3 μm, as shown in Figure A4a. Then, use the HF to etch the step with 1.5–2.5 μm depth; and error for the pattern dimension was ±0.5 μm, as shown in Figure A4b. Finally, the via hole at the center of the glass base was fabricated by liquid blasting method.
